# Accuracy of wearable smartwatch for measuring blood pressure and oxygen saturation across a wide altitudinal gradient: a comparative study in Migrant and Resident populations

**DOI:** 10.3389/fphys.2026.1746894

**Published:** 2026-02-02

**Authors:** Runhao Xu, Jiajun Guo, Weihao Li, Zimo Xie, Yujia Zhai, Siyuan Jiang, Yue Li, Kailun Xia, Yucheng Chen

**Affiliations:** 1 Department of Cardiology, West China Hospital, Sichuan University, Chengdu, Sichuan, China; 2 Huawei Device Co., Ltd, Shenzhen, China; 3 Cardiac Imaging and Target Therapy Lab, West China Hospital, Sichuan University, Chengdu, Sichuan, China; 4 Frontiers Medical Center, Tianfu Jincheng Laboratory, Chengdu, Sichuan, China

**Keywords:** altitude, blood pressure determination, ethnicity, oximetry, wearable electronic devices

## Abstract

**Background:**

Wearable devices are increasingly used to monitor physiological parameters, yet their accuracy at high altitude remains uncertain. We evaluated a commercial smartwatch for peripheral oxygen saturation (SpO_2_) and blood pressure (BP) measurement across a wide altitudinal gradient and examined whether adaptation status and ethnicity influence accuracy.

**Methods:**

A prospective observational study was conducted at four sites in Western China (500–4,014 m). The Migrant cohort (n = 24) comprised lowlanders assessed longitudinally at all sites; the Resident cohort (n = 85) comprised long-term high-altitude residents sampled cross-sectionally. Smartwatch measurements (HUAWEI Watch GT3 Pro) were compared with reference devices using Bland–Altman analysis and benchmarked against ANSI/AAMI/ISO standards.

**Results:**

SpO_2_ accuracy was maintained across altitudes, with RMSE of 0.19%–0.81%, within the ISO 80601-2-61:2017 threshold (≤3%). BP showed wider limits of agreement with a persistent negative bias. SBP in Migrants at 2,560 m exceeded ISO 81060-2:2018 criteria for both bias (−5.29 mmHg) and SD (9.88 mmHg; threshold ≤8 mmHg). DBP bias in Migrants at 4,014 m also exceeded the ±5 mmHg threshold (−5.28 mmHg). No significant differences in measurement error were found between Migrants and Residents or between Han and Zang ethnicities after FDR correction, though error variability was markedly higher in acutely exposed individuals (variance ratio up to 17.5 for SpO_2_).

**Conclusion:**

Smartwatch SpO_2_ readings remained within ISO benchmark thresholds, supporting trend monitoring utility. BP readings showed specific deviations from ISO criteria during acute exposure and warrant cautious interpretation above 2,500 m.

## Introduction

Wearable technologies, particularly smartwatches that employ photoplethysmography (PPG), are increasingly used for out-of-clinic physiological monitoring (Charlton et al., 2022; Bayoumy et al., 2021; Sastimoglu et al., 2025; Jasinski et al., 2024). By providing continuous, non-invasive estimates of peripheral oxygen saturation (SpO_2_) and blood pressure (BP), these devices have potential utility in remote care, early risk detection, and self-management (Kario, 2020; Zhao et al., 2023; Islam et al., 2022; Prieto-Avalos et al., 2022). Their clinical value, however, depends on robust validation under conditions in which accurate monitoring is most needed (Bayoumy et al., 2021; Izmailova et al., 2018; Wen et al., 2017; Goldsack et al., 2020). In physiologically challenging environments, consumer-grade algorithms may not generalize, and measurement error may be amplified (Nelson et al., 2020; Ates et al., 2022; Alam and Hamida, 2014).

High altitude is a stringent natural stressor that induces hypobaric hypoxia and complex cardiorespiratory adjustments, including increased heart rate and cardiac output, peripheral vasoconstriction with attendant BP changes, and progressive declines in SpO_2_ (Naeije, 2010; Mallet et al., 2021; Riley and Gavin, 2017; Bärtsch and Gibbs, 2007). Such perturbations, together with reduced skin perfusion and temperature, can degrade PPG signal quality and confound algorithmic estimation of SpO_2_ and BP (Castiglioni et al., 2022; Nardelli and Bailón, 2023). Most commercial algorithms have been developed and validated near sea level in healthy populations, raising concerns about performance at altitude (Dünnwald et al., 2021; Rafl et al., 2022). Although prior studies have examined the accuracy of wearables (Huhn et al., 2022; Jamieson et al., 2025), comprehensive evaluations across a broad altitudinal gradient and in diverse human populations are scarce.

Physiological responses to altitude also vary by adaptive state. Individuals acutely exposed to high altitude (e.g., lowlanders traveling to altitude) exhibit a volatile, stress-related physiology, whereas lifelong residents at high altitude manifest stable, long-term adaptations (Moore, 2017; Sharma et al., 2022). Genetic factors further contribute to heterogeneity; for example, Tibetan (Zang) populations harbor variants (e.g., in EPAS1) associated with improved hypoxic tolerance relative to Han populations living at similar elevations (Shi et al., 2023; Moya et al., 2024; Li et al., 2019). Whether smartwatch accuracy is differentially affected by acute versus chronic adaptation, or by ethnicity within chronically adapted residents, remains inadequately characterized (Ye et al., 2023; Zeng et al., 2024).

Accordingly, we conducted a comprehensive evaluation of a commercially available smartwatch against gold-standard clinical methods across four sites spanning 500–4,014 m. Our objectives were to (Charlton et al., 2022) quantify how measurement accuracy for SpO_2_ and BP changes with increasing altitude (Bayoumy et al., 2021); compare measurement error between lowlanders undergoing acute exposure (Migrant group) and high-altitude residents with chronic adaptation (Resident group); and (Sastimoglu et al., 2025) explore whether accuracy differs between Han and Zang ethnicities within the Resident group. We hypothesized that accuracy would deteriorate with increasing altitude and that errors would be greater in acutely exposed lowlanders than in chronically adapted residents.

## Methods

### Study design and setting

**FIGURE 1:**
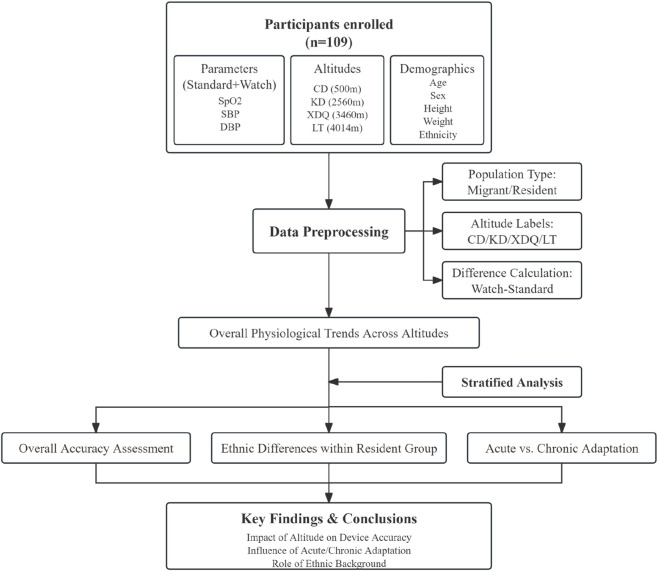
Flow diagram of the study design and participant analysis. The workflow illustrates the process from initial data collection to final stratified analysis. Data on three physiological parameters (SpO_2_, SBP, DBP) were collected from participants at four distinct altitudes. Participants were categorized into Migrant (acute high-altitude exposure) and Resident (chronic high-altitude exposure) groups. Subsequent analyses compared measurement accuracy between these groups and further explored ethnic differences (Han vs. Zang) within the Resident group. Abbreviations: SpO_2_, peripheral oxygen saturation; SBP, systolic blood pressure; DBP, diastolic blood pressure; CD, Chengdu; KD, Kangding; XDQ, Xinduqiao; LT, Litang.

We conducted a prospective observational study that integrated a longitudinal component with parallel cross-sectional cohorts. Data were collected at four sites spanning a broad altitudinal gradient in Western China: Chengdu (CD, 500 m), Kangding (KD, 2,560 m), Xinduqiao (XDQ, 3,460 m), and Litang (LT, 4,014 m). This study was prospectively registered in the Chinese Clinical Trial Registry (registration number: ChiCTR2100048780) and was approved by the Institutional Review Board of West China Hospital. All procedures were performed in accordance with the Declaration of Helsinki. Written informed consent was obtained from all participants prior to any study procedures. An overview of the study workflow and sampling scheme is shown in Figure 1.

### Study population

Participants were enrolled into two prespecified cohorts. The Migrant cohort comprised healthy lowlanders permanently residing at the 500 m site who underwent baseline assessment in Chengdu and subsequent repeated measurements at KD, XDQ, and LT to model acute high-altitude exposure. The Resident cohort comprised three independent cross-sectional samples of healthy long-term residents recruited locally at KD, XDQ, and LT to represent chronic adaptation to altitude. Eligible participants were 18–60 years old and willing to provide informed consent. Individuals were excluded if they reported cardiovascular, respiratory, or hematologic disease, current pregnancy, or regular use of medications known to affect cardiovascular function. Demographic data included age, sex, height, weight, and self-reported ethnicity (Han, Zang, or Yi).

### Data collection and procedures

All measurements were obtained in a quiet, temperature-controlled room (20 °C–25 °C) after at least 10 min of seated rest. Each session included paired assessments using a commercially available smartwatch and reference medical devices performed in close temporal succession. The smartwatch (HUAWEI Watch GT3 Pro) employs photoplethysmography to estimate peripheral SpO_2_, systolic blood pressure (SBP), and diastolic blood pressure (DBP). Importantly, the GT3 Pro model used in this study was equipped with the TruSeen™ 5.0+ optical heart rate monitoring system with enhanced PPG sensors. For blood pressure estimation, a research-grade algorithm provided by Huawei for investigational purposes was enabled through a research collaboration; this algorithm is distinct from the consumer-facing features available on commercially marketed HUAWEI Watch GT3 Pro devices and should not be conflated with standard retail functionality. The device was worn snugly on the non-dominant wrist following the manufacturer’s guidance. Measurements were performed under controlled ambient lighting conditions with overhead fluorescent illumination and no direct sunlight. Watch band tightness was adjusted to allow one finger to fit snugly between the band and the wrist, ensuring secure contact without excessive compression of underlying tissues. Reference SpO_2_ was measured with a medical-grade finger pulse oximeter placed on the non-dominant index finger. Reference SBP and DBP were obtained by a trained operator using a mercury sphygmomanometer and stethoscope (auscultatory method) on the non-dominant arm. Cuff size was selected according to the participant’s mid-arm circumference following international guidelines, with the bladder encircling at least 80% of the arm (See details in Supplementary Figure S1). Two consecutive readings were obtained at least 1 minute apart; if the difference between them exceeded 5 mmHg for either SBP or DBP, another two paired measurements would be taken until the difference was within 5 mmHg. The time interval between corresponding smartwatch and reference device measurements was kept within 2 minutes to minimize the influence of short-term physiological fluctuations. Participants were seated quietly with feet flat on the floor and the arm supported at heart level throughout all measurements.

### Variables and Definitions

Participants permanently residing at 500 m were classified as Migrants, whereas those with long-term residence at 2,560 m, 3,460 m, or 4,014 m were classified as Residents. For each parameter, measurement error was defined as the smartwatch value minus the corresponding reference value, such that negative values indicate underestimation by the smartwatch.

### Data quality control

Prior to analysis, a data quality check was performed in accordance with the operational requirements of ANSI/AAMI/ISO 81060-2:2018 (ISO 81060-2: 2018 S nOf, 2018). For each participant at each altitude, we examined the consistency of the two reference blood pressure readings. Sessions with >5 mmHg difference between duplicate reference BP readings were flagged for exclusion. No sessions met this criterion (0/181 flagged), indicating high reference measurement consistency.

### Statistical analysis

All analyses were performed in Python (version 3.10). Two-sided P-values <0.05 were considered statistically significant. Baseline characteristics were summarized as mean ± standard deviation (SD) for continuous variables and as counts and percentages for categorical variables, with group differences examined using one-way analysis of variance or the chi-square test, as appropriate.

Agreement between smartwatch and reference measurements was evaluated with Bland–Altman methods, reporting the mean difference (bias) and the 95% limits of agreement, calculated as bias ±1.96 times the SD of the paired differences. To quantify absolute error magnitude, we also computed the mean absolute error (MAE) and the root mean square error (RMSE) within each subgroup.

Confounder Assessment. Given the imbalanced sex distribution observed at 3,460 m (23.3% male), we assessed whether sex was a potential confounder for measurement error. At this altitude, independent-samples t-tests were used to compare the mean error (Watch − Standard) between male and female participants for each physiological parameter. For DBP, a statistically significant association between sex and measurement error was identified (P = 0.029). Consequently, all subsequent analyses involving DBP error employed sex-adjusted residuals derived from a linear regression model (DBP Error ∼ Sex), ensuring that the primary comparisons (e.g., Migrant vs. Resident, Han vs. Zang) were not confounded by the unequal sex distribution. For SpO_2_ and SBP, no significant sex effect was detected (P = 0.73 and P = 0.17, respectively), and unadjusted errors were used.

Benchmarking Against International Standards. We benchmarked device performance against relevant international standards. For blood pressure, we assessed compliance with the ANSI/AAMI/ISO 81060-2:2018 Universal Standard (including Amd 1:2020) (EN, 2011) using its two validation criteria. Criterion 1 requires that, across all paired determinations, the absolute mean difference does not exceed 5 mmHg and the SD does not exceed 8 mmHg. Criterion 2 requires that the SD of the per-subject mean differences remains below a bias-dependent limit, which is approximately 6.9 mmHg when the overall bias is near zero. Both criteria were calculated within each altitude stratum and for the combined dataset to contextualize accuracy across conditions.

For oxygen saturation, we compared RMSE values with the accuracy requirement specified in ANSI/AAMI/ISO 80601-2-61:2017(33) for pulse oximeters, which summarizes accuracy using the root-mean-square error (A_rms) across the SaO_2_ range of 70%–100%, with a pass threshold of A_rms ≤3%. Because arterial co-oximetry was not performed in this field study, RMSE served as a pragmatic proxy for A_rms relative to a medical-grade finger oximeter; compliance with the standard is therefore interpreted as benchmarking rather than formal certification.

Hypothesis Testing and Multiple Comparison Correction. Hypothesis testing followed the study design. Within the longitudinal Migrant cohort, we assessed whether the distribution of measurement error changed across the four altitudes using the Friedman test for repeated measures.

At each high-altitude site, mean errors were compared between Migrants and Residents using Welch’s t-test, which does not assume equal variances and is robust to unequal sample sizes. Within the Resident cohort, ethnic differences (Han vs. Zang) were explored at each altitude. Given the small sample size of Han participants at the highest altitude (n = 7 at 4,014 m), the Mann-Whitney U test was additionally employed as a sensitivity analysis to verify the robustness of the parametric findings.

To quantify the magnitude of group differences independent of sample size, Cohen’s d was calculated for each comparison. Effect sizes were interpreted as small (|d| < 0.5), medium (0.5 ≤ |d| < 0.8), or large (|d| ≥ 0.8).

Because multiple comparisons were performed across three parameters (SpO_2_, SBP, DBP) and three high-altitude sites, all P-values from pairwise group comparisons were adjusted for multiple testing using the Benjamini-Hochberg procedure to control the false discovery rate (FDR) at 5%. Adjusted P-values (P_adj) are reported alongside raw values where relevant.

## Results

### Participant characteristics

Across the four altitudes, 181 measurement sessions were obtained. Following data quality screening per ISO 81060-2:2018 requirements (reference BP measurement consistency check), all 181 sessions were retained for analysis (0 exclusions). The Migrant cohort included 24 healthy lowlanders (14/24 male, 58.3%; mean age 31.50 ± 7.53 years) assessed at all sites. The Resident cohort comprised three independent samples totaling 85 participants: 30 at 2,560 m (16/30 male, 53.3%; mean age 45.87 ± 10.86 years), 30 at 3,460 m (7/30 male, 23.3%; mean age 33.77 ± 7.78 years), and 25 at 4,014 m (10/25 male, 40.0%; mean age 37.04 ± 10.06 years). The ethnic composition of Residents shifted with altitude, with a greater proportion of Zang participants at higher elevations. Detailed demographics are provided in Table 1.

**TABLE 1:** Baseline demographic characteristics of study participants. Continuous variables are presented as mean ± standard deviation (SD). Categorical variables are presented as count (n) and percentage (%).

Demographic characteristics		Resident altitude			
		500 m (n = 24)	2560 m (n = 30)	3460 m (n = 30)	4014 m (n = 25)
Sex n (%)	Male	14 (58.3%)	16 (53.3%)	7 (23.3%)	10 (40.0%)
	Female	10 (41.7%)	14 (46.7%)	23 (76.7%)	15 (60.0%)
Age (y)		31.50 ± 7.53	45.87 ± 10.86	33.77 ± 7.78	37.04 ± 10.06
Height (cm)		167.21 ± 6.59	165.97 ± 8.15	162.70 ± 6.39	166.76 ± 8.83
Weight (kg)		68.02 ± 11.55	67.90 ± 11.82	61.22 ± 12.54	66.26 ± 12.01
Race n (%)	Han	24 (100%)	15 (50.0%)	11 (36.7%)	7 (28.0%)
	Zang	0	15 (50.0%)	18 (60.0%)	18 (72.0%)
	Yi	0	0	1 (3.3%)	0

### Physiological trends and device measurements

**FIGURE 2:**
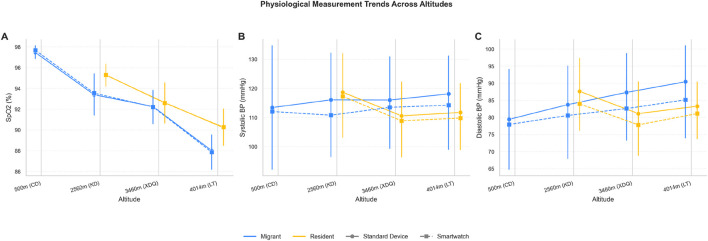
Trends in physiological measurements across altitudes for Migrant and Resident groups. Line plots show the mean values (±SD, indicated by error bars) for **(A)** peripheral oxygen saturation (SpO_2_), **(B)** systolic blood pressure (SBP), and **(C)** diastolic blood pressure (DBP) measured at four altitudes. Solid lines with circular markers represent measurements from the standard medical-grade reference devices, while dashed lines with square markers represent measurements from the wearable smartwatch. Blue lines indicate the Migrant group (lowlanders undergoing acute high-altitude exposure), and yellow/orange lines indicate the Resident group (long-term high-altitude residents representing chronic adaptation). As altitude increased, SpO_2_ declined progressively in both groups **(A)**, with the Migrant group showing a steeper decline and lower absolute values compared to the Resident group. Blood pressure showed altitude-dependent changes **(B,C)**, with the Migrant group exhibiting higher SBP and DBP than the Resident group at high-altitude sites. The wearable device tracked these physiological trends closely across all conditions. Abbreviations: SpO_2_, peripheral oxygen saturation; SBP, systolic blood pressure; DBP, diastolic blood pressure; SD, standard deviation; CD, Chengdu (500 m); KD, Kangding (2,560 m); XDQ, Xinduqiao (3,460 m); LT, Litang (4,014 m).

As altitude increased, standard measurements showed a progressive decline in SpO_2_ and a general increase in blood pressure, particularly in the Migrant group (Table 2; Figures 2A–C). The wearable device successfully tracked these physiological trends for SpO_2_, SBP, and DBP in both Migrant and Resident groups (Figures 2A–C). At all high-altitude sites, the Resident group maintained a higher mean SpO_2_ (Figure 2A) and a lower mean SBP (Figure 2B) and DBP (Figure 2C) compared to the Migrant group (Table 2).

**TABLE 2:** Standard and wearable device measurements stratified by altitude and population group. Data are presented as mean ± standard deviation. The table compares physiological measurements obtained from a standard medical-grade device and a wearable device across four altitudes. Data are stratified by population group: the Migrant (M) group, representing individuals with acute high-altitude exposure, and the Resident (R) group, representing individuals with chronic high-altitude exposure. The Combined (C) group includes all participants (both Migrant and Resident) at a given high altitude. Abbreviations: SpO_2_, peripheral oxygen saturation; SBP, systolic blood pressure; DBP, diastolic blood pressure; M, Migrant; R, Resident; C, Combined; n, number of participants.

Measurements		Measure altitude									
		500m	2560m			3460m			4014m		
		M (n = 24)	M (n = 24)	R (n = 30)	C (n = 54)	M (n = 24)	R (n = 30)	C (n = 54)	M (n = 24)	R (n = 25)	C (n = 49)
SpO_2_ (%)	Standard	97.46 ± 0.59	93.40 ± 1.97	95.29 ± 1.07	94.45 ± 1.79	92.26 ± 1.50	92.63 ± 1.90	92.47 ± 1.73	88.01 ± 1.46	90.22 ± 1.69	89.14 ± 1.92
	Watch	97.68 ± 0.44	93.55 ± 1.86	92.23 ± 1.61	94.52 ± 1.68	92.23 ± 1.61	92.60 ± 1.94	92.44 ± 1.80	87.87 ± 1.66	90.27 ± 1.76	89.10 ± 2.08
SBP (mmHg)	Standard	113.43 ± 21.26	116.08 ± 16.06	118.69 ± 13.30	117.53 ± 14.51	116.02 ± 14.85	110.57 ± 11.64	112.99 ± 13.31	118.13 ± 13.05	111.74 ± 9.94	114.87 ± 11.89
	Watch	112.06 ± 16.46	110.79 ± 14.21	117.29 ± 14.10	114.40 ± 14.39	113.50 ± 14.12	108.88 ± 12.38	110.93 ± 13.26	114.25 ± 15.20	109.81 ± 10.84	111.99 ± 13.21
DBP (mmHg)	Standard	79.43 ± 14.63	83.74 ± 11.28	87.65 ± 9.64	85.91 ± 10.48	87.33 ± 11.37	81.12 ± 9.35	83.88 ± 10.66	90.44 ± 10.56	83.28 ± 7.11	86.79 ± 9.58
	Watch	77.94 ± 12.46	80.57 ± 12.60	83.96 ± 7.77	82.45 ± 10.23	82.59 ± 9.25	77.83 ± 8.96	79.95 ± 9.32	85.15 ± 11.14	81.12 ± 7.31	83.09 ± 9.50

### Agreement and error analysis

**FIGURE 3:**
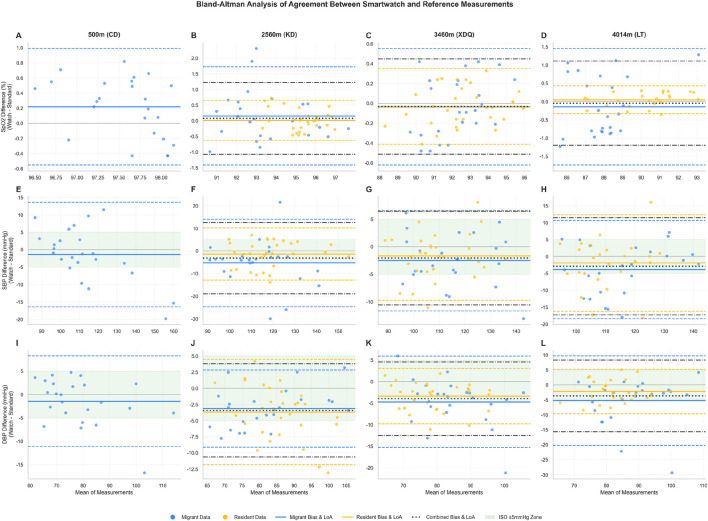
Bland-Altman analysis of agreement between wearable device and standard measurements. Bland-Altman plots illustrating the agreement between measurements from the wearable smartwatch and the standard medical-grade reference device for peripheral oxygen saturation (SpO_2_; panels **(A–D)**, systolic blood pressure (SBP; panels **(E–H)**, and diastolic blood pressure (DBP; panels **(I–L)**). Each column represents a different measurement altitude: 500 m (CD; **(A,E,I)**, 2,560 m (KD; **(B,F,J)**, 3,460 m (XDQ; **(C,G,K)**, and 4,014 m (LT; **(D,H,L)**). The y-axis displays the difference between methods (Watch − Standard), such that negative values indicate underestimation by the smartwatch. Individual data points are color-coded by population group: blue for the Migrant group and yellow for the Resident group. For each group, the solid horizontal line represents the mean difference (bias), and the corresponding dashed lines represent the 95% limits of agreement (LoA), calculated as bias ±1.96 × SD. At high-altitude sites (KD, XDQ, LT), the black dotted line and dash-dotted lines represent the bias and 95% LoA for the combined population (both Migrant and Resident groups pooled), respectively. The green shaded zone in SBP and DBP panels **(E–L)** indicates the ANSI/AAMI/ISO 81060-2:2018 acceptable bias range (±5 mmHg). Notably, the Migrant group at 2,560 m **(F)** exhibited bias and SD values exceeding the ISO thresholds (bias = −5.29 mmHg, SD = 9.88 mmHg), and the Migrant group at 4,014 m **(L)** showed DBP bias exceeding the threshold (bias = −5.28 mmHg). Abbreviations: SpO_2_, peripheral oxygen saturation; SBP, systolic blood pressure; DBP, diastolic blood pressure; LoA, limits of agreement; SD, standard deviation; CD, Chengdu (500 m); KD, Kangding (2,560 m); XDQ, Xinduqiao (3,460 m); LT, Litang (4,014 m).

Bland–Altman analysis demonstrated that agreement between the smartwatch and reference devices varied by parameter and altitude (Figure 3). For SpO_2_ (Figures 3A–D), agreement at 500 m was high, with a narrow 95% LoA (−0.55%–0.99%) and low MAE (0.40%) (Table 3). At higher altitudes, LoA widened and both MAE and RMSE increased, particularly in Migrants at 4,014 m (MAE 0.75%, RMSE 0.81%; Figure 3D). Notably, across all sites and groups the SpO_2_ RMSE ranged from 0.19% to 0.81%, which is within the ANSI/AAMI/ISO 80601-2-61:2017 accuracy requirement for pulse oximeters (A_rms ≤3%).

**TABLE 3:** Error metrics for wearable device measurements stratified by altitude and population group. Data are presented for each physiological parameter. Bias is the mean difference (Watch - Standard). SD represents the standard deviation of mean differences per subject (ISO 81060–2 Criterion 2). Bold values indicate instances exceeding ISO 81060-2:2018 thresholds (|Bias| > 5 mmHg or SD > 8 mmHg). LoA (95% Limits of Agreement) is the interval [Bias - 1.96 × SD, Bias +1.96 × SD], where SD is the standard deviation of the difference. MAE (Mean Absolute Error) is the average of the absolute differences. RMSE (Root Mean Square Error) is the square root of the average of the squared differences. Data are stratified by population group: the Migrant (M) group, representing individuals with acute high-altitude exposure, and the Resident (R) group, representing individuals with chronic high-altitude exposure. The Combined (C) group includes all participants (both Migrant and Resident) at a given high altitude. Abbreviations: SpO_2_, peripheral oxygen saturation; SBP, systolic blood pressure; DBP, diastolic blood pressure; M, Migrant; R, Resident; C, Combined; n, number of participants.

Measurements		Measure altitude									
		500m	2560m			3460m			4014m		
		M (n = 24)	M (n = 24)	R (n = 30)	C (n = 54)	M (n = 24)	R (n = 30)	C (n = 54)	M (n = 24)	R (n = 25)	C (n = 49)
SpO_2_ (%)	Bias	0.22	0.15	0.01	0.08	−0.03	−0.03	−0.03	−0.14	0.05	−0.05
	SD	0.39	0.80	0.33	0.59	0.30	0.20	0.24	0.81	0.19	0.59
	LoA	(-0.55, 0.99)	(-1.42, 1.72)	(-0.63, 0.66)	(-1.07, 1.22)	(-0.62, 0.55)	(-0.41, 0.35)	(-0.51, 0.45)	(-1.74, 1.45)	(-0.33, 0.43)	(-1.20, 1.11)
	MAE	0.40	0.60	0.25	0.41	0.26	0.16	0.21	0.75	0.17	0.45
	RMSE	0.44	0.80	0.32	0.58	0.30	0.19	0.24	0.81	0.20	0.58
SBP (mmHg)	Bias	−1.38	−5.29	−1.40	−3.13	−2.52	−1.69	−2.06	−3.88	−1.92	−2.88
	SD	7.66	**9.88**	5.87	**8.06**	4.65	4.10	4.33	7.41	7.30	7.35
	LoA	(-16.40, 13.64)	(-24.65, 14.07)	(-12.92, 10.11)	(-18.93, 12.67)	(-11.64, 6.60)	(-9.73, 6.35)	(-10.55, 6.43)	(-18.41, 10.65)	(-16.24, 12.39)	(-17.28, 11.52)
	MAE	5.85	7.78	4.78	6.11	4.25	3.36	3.76	6.46	5.22	5.83
	RMSE	7.63	11.02	5.94	8.58	5.21	4.37	4.76	8.23	7.41	7.82
DBP (mmHg)	Bias	−1.48	−3.18	−3.69	−3.46	−4.74	−3.29	−3.93	**−5.28**	−2.16	−3.69
	SD	4.96	3.07	4.15	3.68	5.46	3.31	4.41	7.61	3.68	6.08
	LoA	(-11.20, 8.23)	(-9.18, 2.83)	(-11.81, 4.44)	(-10.67, 3.75)	(-15.45, 5.97)	(-9.78, 3.20)	(-12.58, 4.72)	(-20.20, 9.63)	(-9.37, 5.05)	(-15.61, 8.23)
	MAE	3.81	3.84	4.34	4.12	5.48	3.57	4.42	5.84	3.65	4.72
	RMSE	5.07	4.37	5.50	5.03	7.15	4.63	5.88	9.13	4.20	7.06

Bold values indicate instances exceeding ISO 81060-2:2018 thresholds (|Bias| > 5 mmHg or SD > 8 mmHg).

For SBP (Figures 3E–H), the measurements demonstrated wider limits of agreement across conditions. Among Migrants, LoA ranged from −16.40 to 13.64 mmHg at 500 m (Figure 3E) to −18.41 to 10.65 mmHg at 4,014 m (Figure 3H). The smartwatch showed a persistent negative bias (underestimation), most pronounced in Migrants at 2,560 m (bias −5.29 mmHg, SD 9.88 mmHg; Figure 3F), which exceeded both the mean-bias threshold (|bias| ≤ 5 mmHg) and the SD threshold (≤8 mmHg) specified by ANSI/AAMI/ISO 81060-2:2018 Criterion 1. Additionally, the Combined group at 2,560 m exhibited an SD of 8.06 mmHg, marginally exceeding the 8 mmHg limit (Table 3).

For DBP (Figures 3I–L), the smartwatch also exhibited a consistent negative bias across groups and altitudes. LoA were narrower than for SBP but wider than for SpO_2_. The largest bias was observed in Migrants at 4,014 m (bias −5.28 mmHg, exceeding the ±5 mmHg threshold; Figure 3L), with an SD of 7.61 mmHg that approached but did not exceed the 8 mmHg limit (Table 3).

In summary, when benchmarked against ANSI/AAMI/ISO 81060-2:2018, the smartwatch met the mean-bias criterion (|bias| ≤ 5 mmHg) in most strata but showed three specific instances of non-compliance (Table 3, bolded values): (Charlton et al., 2022) SBP in Migrants at 2,560 m exceeded both bias and SD thresholds (Bayoumy et al., 2021); Combined SBP at 2,560 m exceeded the SD threshold; and (Sastimoglu et al., 2025) DBP in Migrants at 4,014 m exceeded the bias threshold. These findings indicate that measurement consistency, particularly for SBP under acute high-altitude exposure, did not fully align with the standard’s requirements.

### Effect of acute high-altitude exposure on measurement error

**FIGURE 4:**
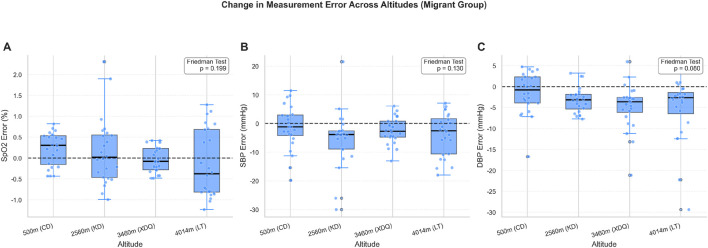
Longitudinal analysis of measurement error in the Migrant group across altitudes. Boxplots illustrate the distribution of measurement error (Watch − Standard) for **(A)** SpO_2_, **(B)** SBP, and **(C)** DBP in the same cohort of Migrant participants (n = 24) measured longitudinally at four different altitudes during their ascent from 500 m to 4,014 m. The central line indicates the median, the box represents the interquartile range (IQR, 25th–75th percentile), and the whiskers extend to 1.5 times the IQR. Individual data points are overlaid as dots to show the full distribution. The horizontal dashed line at zero indicates perfect agreement (no bias). P values in the upper right corner of each panel were derived from the Friedman test, a non-parametric test for repeated measures, to assess whether the distribution of measurement error changed significantly across the four altitudes within this longitudinal cohort. No statistically significant change was observed for SpO_2_ (P = 0.199) or SBP (P = 0.130), while DBP showed a trend toward increasing negative bias (underestimation) with altitude that approached but did not reach statistical significance (P = 0.080). Abbreviations: SpO_2_, peripheral oxygen saturation; SBP, systolic blood pressure; DBP, diastolic blood pressure; IQR, interquartile range; CD, Chengdu (500 m); KD, Kangding (2,560 m); XDQ, Xinduqiao (3,460 m); LT, Litang (4,014 m).

A longitudinal analysis of the Migrant group showed no statistically significant change in the distribution of measurement error for SpO_2_ (Friedman test, P = 0.199; Figure 4A) or SBP (P = 0.130; Figure 4B) across the four altitudes. However, a trend towards a more negative bias in DBP error was observed with increasing altitude, although this did not reach statistical significance (P = 0.080; Figure 4C).

**FIGURE 5:**
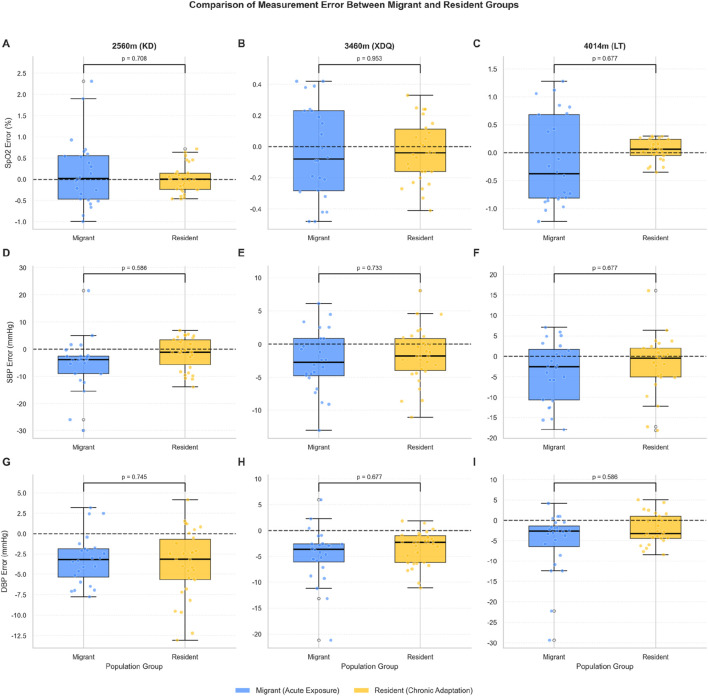
Comparison of measurement error between Migrant (acute exposure) and Resident (chronic adaption) groups at high altitudes. Boxplots compare the measurement error (Watch − Standard) for SpO_2_ **(A–C)**, SBP **(D–F)**, and DBP **(G–I)** between Migrant and Resident groups at three high altitudes: 2,560 m (KD; **(A,D,G)**, 3,460 m (XDQ; **(B,E,H)**, and 4,014 m (LT; **(C,F,I)**. For DBP, sex-adjusted residuals were used to account for the confounding effect of sex identified at 3,460 m. The central line indicates the median, the box represents the interquartile range (IQR), and whiskers extend to 1.5 times the IQR. Individual data points are overlaid. The horizontal dashed line at zero indicates perfect agreement (no bias). P-values were calculated using Welch’s t-test and adjusted for multiple comparisons using the Benjamini-Hochberg procedure to control the false discovery rate; adjusted P-values (P_adj) are displayed. At 4,014 m, despite non-significant differences in mean error, the Migrant group exhibited substantially greater variability in SpO_2_ measurements (variance ratio = 17.5), as reflected by the wider distribution in panel **(C)**. Abbreviations: SpO_2_, peripheral oxygen saturation; SBP, systolic blood pressure; DBP, diastolic blood pressure; KD, Kangding; XDQ, Xinduqiao; LT, Litang; IQR, interquartile range.

When comparing the Migrant group to the Resident group at each high altitude (Figure 5), no statistically significant differences were found in the mean measurement error for SpO_2_ or SBP at any altitude after FDR correction (all P_adj >0.05; Figures 5A–F). For DBP (sex-adjusted), a trend towards a more negative error in the Migrant group was observed at 4,014 m (Figure 5I), but this was not statistically significant after correction (raw P = 0.086, P_adj = 0.586).

Notably, while mean errors did not differ significantly between groups, the variability of errors showed a marked disparity. At 4,014 m, the variance ratio (Migrant/Resident) for SpO_2_ error was 17.5, indicating substantially greater measurement variability in the acutely exposed group despite similar mean bias (Figure 5C). This is further reflected in the wider interquartile range observed in the Migrant group. Effect size analysis revealed a medium effect for DBP (Cohen’s d = −0.51), suggesting a potentially meaningful difference that may have been underpowered to detect given the sample sizes. For SpO_2_ and SBP, effect sizes were small (Cohen’s d = −0.33 and −0.27, respectively).

### Effect of ethnicity on measurement error

**FIGURE 6:**
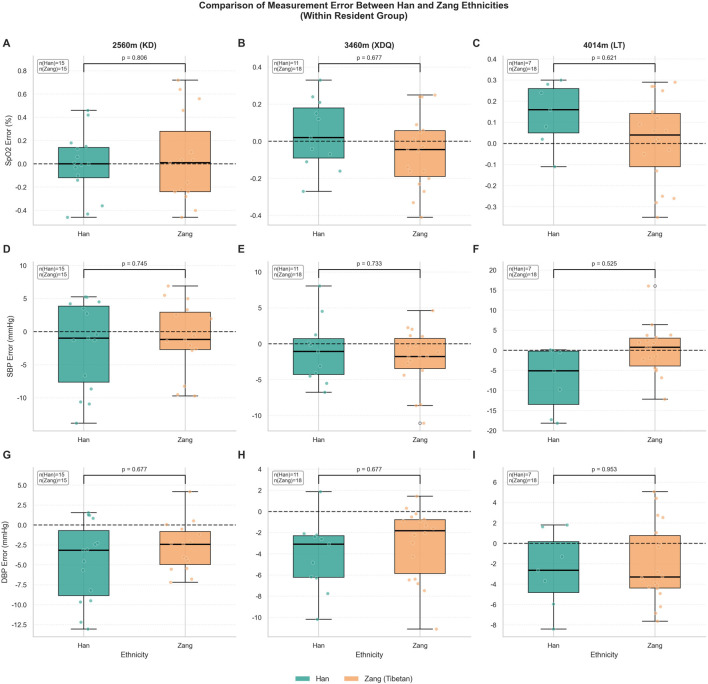
Comparison of measurement error between Han and Zang ethnicities within the Resident group at high altitudes. Boxplots compare the measurement error (Watch − Standard) for SpO_2_ **(A–C)**, SBP **(D–F)**, and DBP **(G–I)** between participants of Han and Zang ethnicity within the chronically adapted Resident group at three high altitudes: 2,560 m (KD; **(A,D,G)**, 3,460 m (XDQ; **(B,E,H)**, and 4,014 m (LT; **(C,F,I)**. For DBP, sex-adjusted residuals were used to account for the confounding effect of sex. The central line indicates the median, the box represents the interquartile range (IQR), and whiskers extend to 1.5 times the IQR. Individual data points are overlaid. The horizontal dashed line at zero indicates perfect agreement (no bias). Sample sizes for each ethnic group at each altitude are displayed in inset annotations (e.g., n (Han) = 7, n (Zang) = 18 at 4,014 m). P-values were calculated using Welch’s t-test; for comparisons involving small samples (n < 10), the Mann-Whitney U test was additionally employed as a sensitivity analysis, yielding consistent results. All P-values were adjusted for multiple comparisons using the Benjamini-Hochberg procedure; adjusted P-values (P_adj) are displayed. Abbreviations: SpO_2_, peripheral oxygen saturation; SBP, systolic blood pressure; DBP, diastolic blood pressure; KD, Kangding; XDQ, Xinduqiao; LT, Litang; IQR, interquartile range.

Within the chronically adapted Resident group, there were no statistically significant differences in measurement error for SpO_2_ or DBP (sex-adjusted) between participants of Han and Zang ethnicity at any of the high-altitude sites after FDR correction (all P_adj >0.05; Figure 6). Sample sizes for Han participants were limited at higher altitudes (n = 15 at 2,560 m, n = 11 at 3,460 m, n = 7 at 4,014 m; Figure 6, inset annotations), which may have reduced statistical power to detect small differences. Sensitivity analyses using the Mann-Whitney U test yielded consistent results with parametric tests.

For SpO_2_ (Figures 6A–C), mean errors were comparable between Han and Zang participants across all altitudes (P_adj = 0.81, 0.68, and 0.62 at 2,560 m, 3,460 m, and 4,014 m, respectively). For SBP (Figures 6D–F), a trend towards a difference in mean error was observed at 4,014 m, with the device tending to underestimate in Han participants and show less bias in Zang participants, but this did not reach statistical significance after correction (raw P = 0.056, P_adj = 0.525; Figure 6F). For DBP (Figures 6G–I), errors were similar between ethnic groups at all altitudes (all P_adj >0.67).

## Discussion

We evaluated a commercial smartwatch for SpO_2_, SBP, and DBP across a broad altitudinal gradient, explicitly contrasting acute and chronic high-altitude adaptation. Three principal findings emerged (Charlton et al., 2022) accuracy was parameter-dependent, with SpO_2_ substantially outperforming blood pressure (Bayoumy et al., 2021); accuracy degraded with increasing altitude, most notably in acutely exposed lowlanders; and (Sastimoglu et al., 2025) performance was broadly generalizable across ethnicities, though subtle divergence appeared under extreme hypoxic stress.

The device tracked expected physiological trends—declining SpO_2_ and altitude-related differences in BP between Migrants and Residents—supporting its value for longitudinal monitoring. Nevertheless, absolute agreement varied by parameter and environment. When benchmarked to ANSI/AAMI/ISO 80601-2-61:2017, SpO_2_ error remained well within the 3% A_rms threshold across all sites, as reflected by RMSE values of 0.19%–0.81%. This suggests that, in resting conditions, the smartwatch can provide clinically acceptable oxygenation estimates over the 70%–100% range, at least relative to a medical-grade finger oximeter. Our findings align with recent studies evaluating consumer wearables at altitude (Rafl et al., 2022). reported that a commercial smartwatch detected short-term hypoxemia comparably to medical-grade devices under controlled hypoxic conditions, with RMSE values below 2% (Rafl et al., 2022). Similarly (Zeng et al., 2024), found that smartwatch SpO_2_ measurements showed acceptable accuracy for predicting acute mountain sickness (Zeng et al., 2024), though they noted increased variability at extreme altitudes. Our study extends these observations by systematically evaluating performance across a continuous altitudinal gradient and by explicitly comparing acute versus chronic adaptation, providing a more comprehensive assessment of device utility in real-world high-altitude settings.

In contrast, blood pressure measurement accuracy warrants more cautious interpretation. Against ANSI/AAMI/ISO 81060-2:2018 criteria, while mean SBP and DBP biases were often within the acceptable range of ±5 mmHg, the dispersion of errors was frequently large. Specifically, three instances of non-compliance were identified: SBP in Migrants at 2,560 m exceeded both the bias threshold (−5.29 mmHg) and the SD threshold (9.88 mmHg); the Combined group at 2,560 m exceeded the SD threshold (8.06 mmHg); and DBP in Migrants at 4,014 m exceeded the bias threshold (−5.28 mmHg). These findings indicate that the smartwatch showed challenges in consistently meeting the 81,060–2 standard’s criteria for error dispersion, particularly for SBP during acute high-altitude exposure. This observation reinforces the need for careful consideration before substituting cuffless, PPG-based BP estimates for traditional sphygmomanometry, especially in physiologically stressed conditions where algorithmic assumptions may not hold.

The altitude effect was most apparent in the acutely exposed cohort. Error variability for SpO_2_ increased with ascent, consistent with greater signal instability in acute hypoxia. Notably, at 4,014 m, the variance of SpO_2_ error in Migrants was 17.5 times greater than in Residents, despite comparable mean bias between groups. This marked disparity in measurement precision rather than accuracy explains the apparent paradox wherein mean errors did not differ significantly between groups yet the MAE in Migrants was over four times that in Residents. Effect size analysis further supported this interpretation: Cohen’s d for DBP indicated a medium effect (−0.51) that may have been underpowered to detect given our sample sizes. These findings underscore that mean bias alone is an incomplete metric of device performance; measurement precision, as reflected in variance and MAE, is equally critical for clinical utility. The increased variability in the acutely exposed group likely reflects the dynamic, stress-related cardiovascular adjustments—including fluctuating peripheral vascular resistance and sympathetic activation—that characterize the acute hypoxic response and degrade PPG signal quality. For DBP specifically, we observed a progressive negative bias among Migrants that approached but did not reach statistical significance at the highest site relative to Residents, suggesting that acute changes in vascular tone and arterial stiffness may perturb algorithmic assumptions embedded in cuffless BP estimation (Mukkamala et al., 2022; Hu et al., 2023; Nachman et al., 2020; Avolio et al., 2022).

Ethnic comparisons within chronically adapted Residents showed no significant differences in SpO_2_ or DBP error after FDR correction, indicating broad algorithmic applicability across Han and Zang participants under stable high-altitude adaptation. However, these null findings should be interpreted with caution given the limited sample sizes, particularly for Han participants at extreme altitudes. The marginal divergence in SBP error at 4,014 m—greater underestimation in Han participants compared with less bias in Zang participants—may reflect subtle, phenotype-level differences in vascular mechanics under severe hypoxia, potentially linked to known high-altitude adaptations in Tibetan populations such as EPAS1 variants affecting vascular tone. Alternatively, this trend could represent a Type II error due to insufficient power. These hypotheses merit targeted physiologic and signal-processing studies with larger, balanced ethnic samples (Boos et al., 2017; Fengler et al., 2022; Herzog et al., 2025).

The practical implications of our findings are twofold. For individuals working or traveling at altitude, the smartwatch can be useful for tracking oxygenation trends and flagging deteriorations in SpO_2_, as our benchmarking indicates accuracy compatible with 80,601-2-61 limits in resting conditions. Trend monitoring, such as detecting a decline of 3%–5% in SpO_2_ during ascent, appears reliable across the altitude range studied, even if absolute values carry some uncertainty at extreme elevations. Conversely, BP readings require more nuanced interpretation that accounts for altitude and adaptive state. At elevations below 2,500 m, SBP and DBP measurements showed acceptable bias and dispersion, suggesting the device may be suitable for general trend monitoring in healthy individuals. Between 2,500 and 3,500 m during acute exposure, increased caution is warranted, particularly for SBP, which showed the largest deviations from ISO criteria; single readings should not be used for clinical decisions in this context. Above 3,500 m or during acute exposure, BP measurements showed sufficient variability that the device cannot be recommended for diagnostic purposes or medication titration, and confirmatory measurements with a validated cuff-based device are advised. Notably, measurement accuracy was generally better in chronically adapted residents than in acutely exposed individuals, suggesting the device may be more reliable for long-term high-altitude populations. These recommendations apply to resting measurements in controlled environments; accuracy during physical exertion or cold exposure remains untested.

This study has notable strengths, including the integration of longitudinal and cross-sectional designs, assessments over a wide altitude range, explicit benchmarking against international performance standards, and rigorous statistical methods including confounder adjustment and multiple comparison correction. Several limitations warrant consideration. Subgroup sample sizes were modest, with a disparity between the Migrant and Resident cohorts that may have limited statistical power, and the small number of Han participants at extreme altitudes reduces the reliability of ethnic comparisons at these sites. The imbalanced sex distribution at 3,460 m was addressed through statistical adjustment for DBP, but unmeasured sex-related factors may still have influenced our findings. We evaluated a single smartwatch model at rest, and performance during motion or cold exposure—both common at altitude—was not assessed. Individual characteristics known to affect PPG signal quality, such as skin pigmentation and wrist circumference, were not systematically evaluated. Additionally, operators were not blinded to previous measurements, introducing the potential for observer bias in reference BP readings. Device calibration stability over repeated measurements and across environmental conditions was not independently verified. Finally, our benchmarking against ISO standards should be interpreted as a pragmatic field comparison rather than formal regulatory validation, as arterial co-oximetry was unavailable for SpO_2_ and the full procedural requirements of the standards were not implemented.

In summary, smartwatch SpO_2_ estimates remained within the ANSI/AAMI/ISO 80601-2-61:2017 accuracy threshold across altitudes in resting conditions, whereas BP estimates did not consistently meet ANSI/AAMI/ISO 81060-2:2018 requirements, particularly for SBP during acute high-altitude exposure. Accuracy was further modulated by altitude and adaptive state, with greater error variability rather than bias in acutely exposed lowlanders—a finding with important implications for interpreting device readings in this population. Future algorithm development should incorporate physiologic context, especially the dynamic vascular changes during acute hypoxia, and be validated prospectively against formal standards with larger, demographically balanced samples to ensure reliability in high-altitude environments and diverse populations.

## Conclusion

This study provides the first systematic field benchmarking of a commercial smartwatch across a continuous altitudinal gradient, comparing acutely exposed lowlanders with chronically adapted highlanders. SpO_2_ accuracy remained within ANSI/AAMI/ISO 80601-2-61:2017 thresholds at all altitudes, supporting its utility for trend monitoring in hypoxic environments. In contrast, blood pressure measurements showed deviations from ISO 81060-2:2018 criteria, particularly for SBP during acute exposure, where both bias and error dispersion exceeded acceptable limits. While these findings represent pragmatic field benchmarking rather than formal regulatory validation, they suggest that smartwatch SpO_2_ monitoring is reliable across altitudes, whereas BP readings warrant cautious interpretation, especially in acutely exposed individuals above 2,500 m.

## Data Availability

The raw data supporting the conclusions of this article will be made available by the authors, without undue reservation.
