# Supplementary material for: Accuracy of wearable smartwatch for measuring blood pressure and oxygen saturation across a wide altitudinal gradient: a comparative study in Migrant and Resident populations
Source: Front Physiol. 2026 Feb 2;17:1746894. doi: 10.3389/fphys.2026.1746894 (PMC12907194; doi:10.3389/fphys.2026.1746894)

**Supplementary Figures**

# **Figure S1.** Illustration of the wearing position of the watch and the placement of the reference devices.

(**A**) Setup for reference SpO2 measurement. The smartwatch is worn with adjusted tightness to ensure secure contact, and a medical-grade finger pulse oximeter is placed on the non-dominant index finger. (**B**) Setup for reference blood pressure measurement. Reference SBP and DBP were obtained by a trained operator using a mercury sphygmomanometer and stethoscope via the auscultatory method on the non-dominant arm.


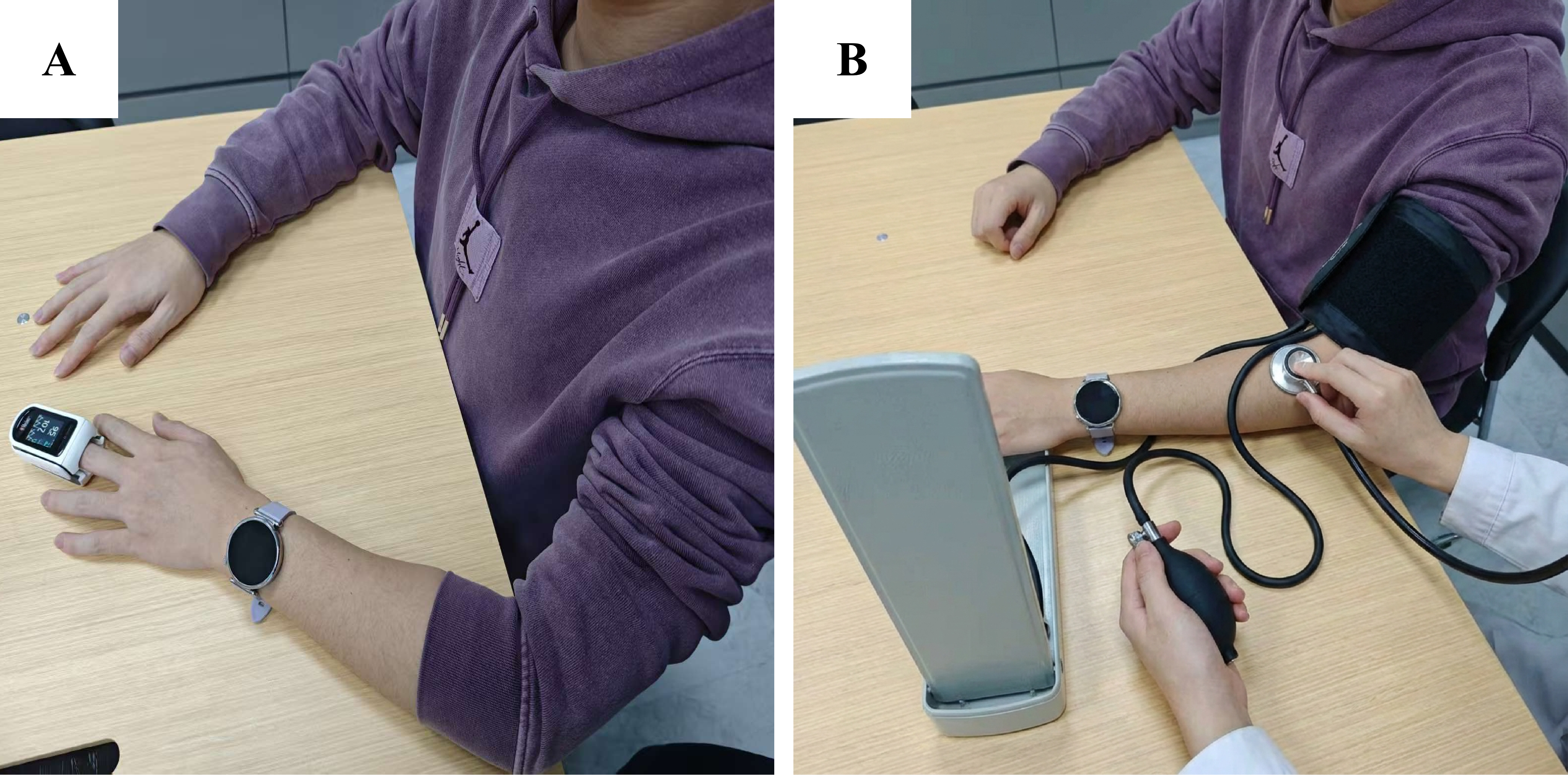


# Figure S2. Correlation analysis between reference and smartwatch blood pressure measurements.

Scatter plots displaying the relationship between reference (x-axis) and smartwatch (y-axis) measurements for (**A**) systolic blood pressure (SBP) and (**B**) diastolic blood pressure (DBP). Data points are color-coded by altitude: blue (500 m, CD), orange (2560 m, KD), green (3460 m, XDQ), and red (4014 m, LT). The black dashed line represents the linear regression fit, and the gray dotted line represents the identity line (perfect agreement). Pearson correlation coefficients (r) and P-values are displayed in each panel. Strong positive correlations were observed for both SBP and DBP across all altitudes, indicating that the smartwatch tracked inter-individual variation in blood pressure effectively despite the systematic bias documented in Table 3.

Abbreviations: SBP, systolic blood pressure; DBP, diastolic blood pressure; CD, Chengdu; KD, Kangding; XDQ, Xinduqiao; LT, Litang.


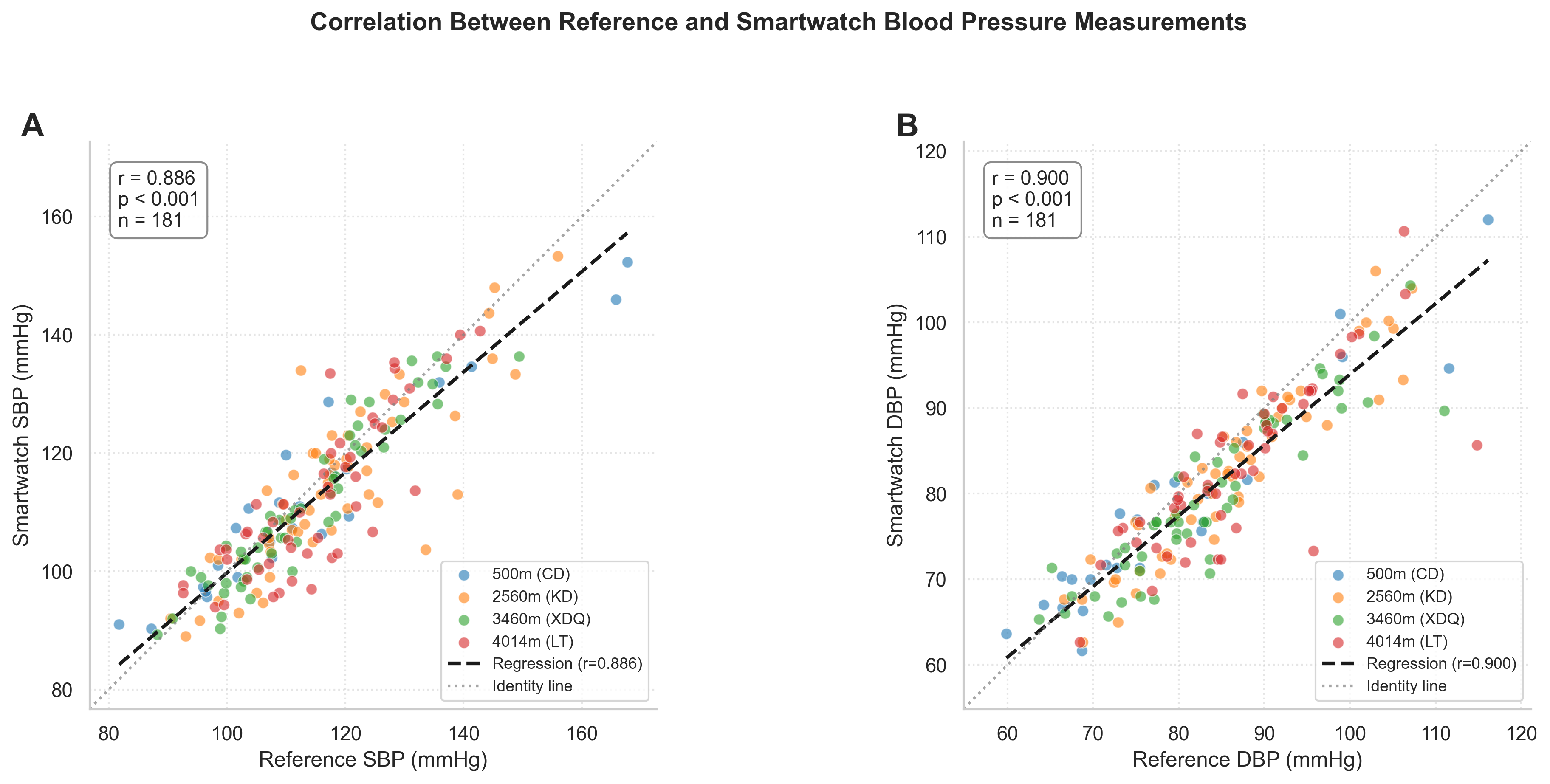

Supplement: Supplementary file 1 [file Supplementaryfile1.docx]
